# Supplementary material for: Murine interfollicular epidermal differentiation is gradualistic with GRHL3 controlling progression from stem to transition cell states
Source: Nat Commun. 2020 Oct 28;11:5434. doi: 10.1038/s41467-020-19234-6 (PMC7595230; doi:10.1038/s41467-020-19234-6)
Supplement: Supplementary file 1 — Supplementary Information [file 41467_2020_19234_MOESM1_ESM.pdf]

Murine Interfollicular epidermal differentiation is gradualistic with GRHL3 controlling progression from stem to transition cell states

Supplementary Informations

Lin et al.

Supplementary Figure 1

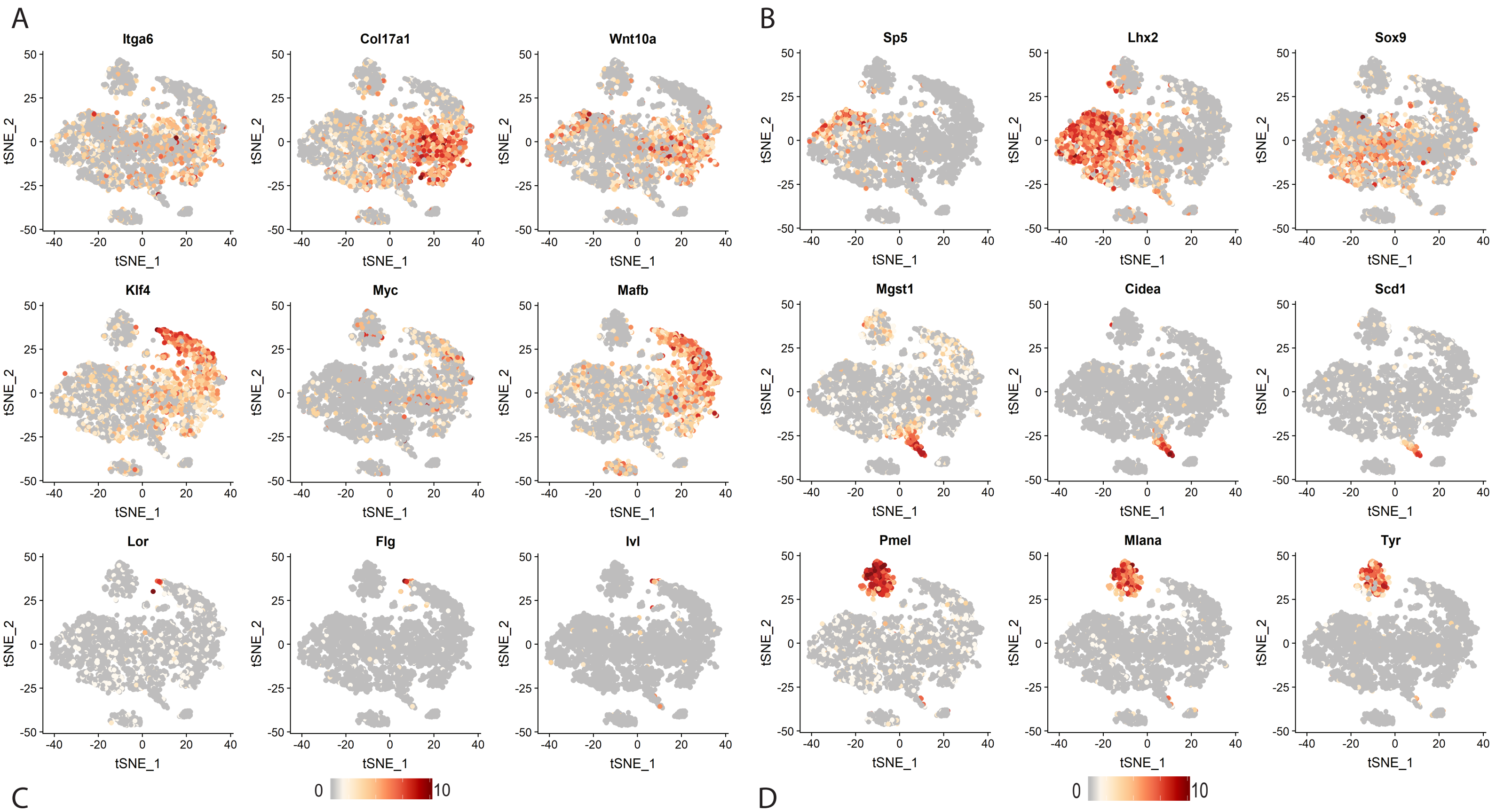

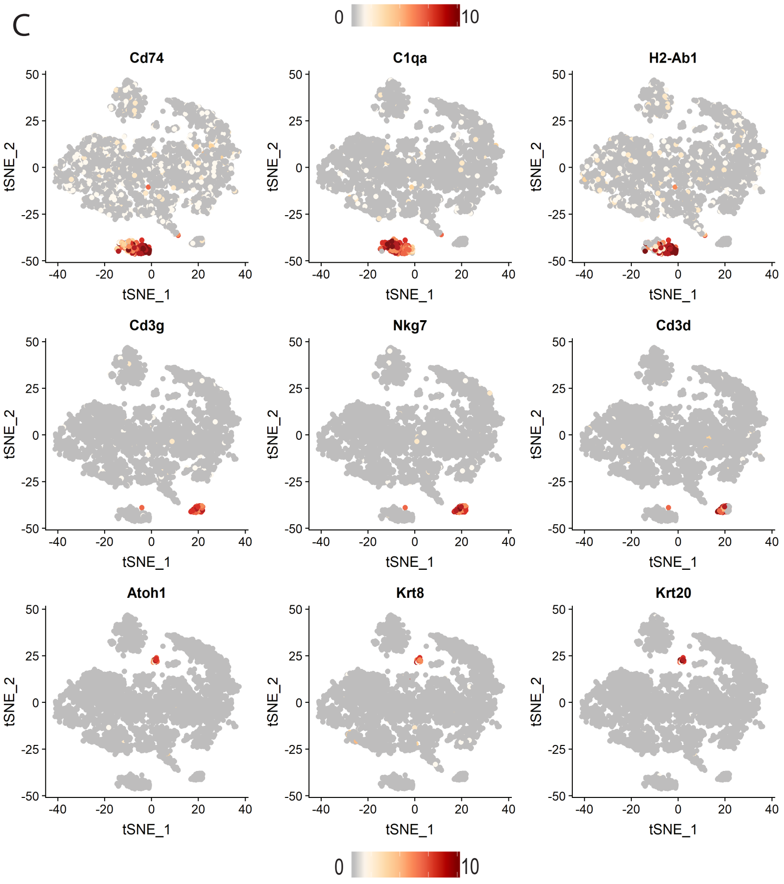

D

GO biological process complete	fold Enrichment	p-value
keratinocyte proliferation (GO:0043616)	48.03	1.52E-02
labyrinthine layer morphogenesis (GO:0060713)	28.91	8.81E-03
skin development (GO:0043588)	7.68	6.20E-04
cell-cell signaling by wnt (GO:0198738)	7.21	4.05E-03
Wnt signaling pathway (GO:0016055)	7.21	4.05E-03
epidermis development (GO:0008544)	6.53	1.06E-02
cell-cell adhesion (GO:0098609)	5.88	3.57E-03
regulation of epithelial cell proliferation (GO:0050678)	5.85	1.06E-02
morphogenesis of an epithelium (GO:0002009)	5.53	1.32E-04

E

GO biological process complete	Fold Enrichment	p-value
keratinocyte differentiation (GO:0030216)	8.91	3.53E-02
epidermal cell differentiation (GO:0009913)	7.38	1.18E-02
skin development (GO:0043588)	6.78	2.55E-04
epidermis development (GO:0008544)	6.74	8.72E-05
epithelium development (GO:0060429)	2.88	3.15E-02
regulation of cell proliferation (GO:0042127)	2.45	2.02E-02
negative regulation of cellular process (GO:0048523)	1.76	2.00E-02
negative regulation of biological process (GO:0048519)	1.7	2.74E-02

F.

GO biological process complete	Fold Enrichment	p-value
desmosome organization (GO:0002934)	27.9	1.09E-02
establishment of skin barrier (GO:0061436)	18.6	1.23E-03
regulation of water loss via skin (GO:0033561)	15.63	3.91E-03
keratinization (GO:0031424)	14.77	1.44E-04
icosanoid biosynthetic process (GO:0046456)	12.6	1.61E-02
water homeostasis (GO:0030104)	10.92	1.81E-03
multicellular organismal water homeostasis (GO:0050891)	10.89	8.73E-03
sphingolipid biosynthetic process (GO:0030148)	8.37	1.59E-02
keratinocyte differentiation (GO:0030216)	6.84	8.48E-04
fatty acid biosynthetic process (GO:0006633)	6.75	9.35E-03

Supplementary Fig. 1. Single cell RNA-seq (scRNA-seq) captures P0 mouse epidermal cell heterogeneity. Related to Figure 1.

A) Expression of canonical basal (*Itga6*, *Col17a1*, *Wnt10a*), differentiated (*Klf4*, *Myc*,*Mafb*), and terminally differentiated (*Lor*, *Flg*, *Ivl*) IFE markers. **B**) Expression of hair follicle (*Sp5*, *Lhx2*, *Sox9*), sebaceous gland (*Mgst1*, *Cidea*, *Scd1*), and melanocyte (*Pmel*, *Mlana*, *Tyr*) markers. **C**) Expression of Langerhans cell (*Cd74*, *C1qa*, *H2-Ab1*), T-cell (*Cd3g*, *Nkg7*, *Cd3d*), and Merkel cell (*Atoh1*, *Krt8*, *Krt20*) markers. **D-F**) GO enrichment categories with fold enrichment and p-value for the marker genes of the basal (D), transition (E), and differentiated (F) IFE populations.

Supplementary Figure 2

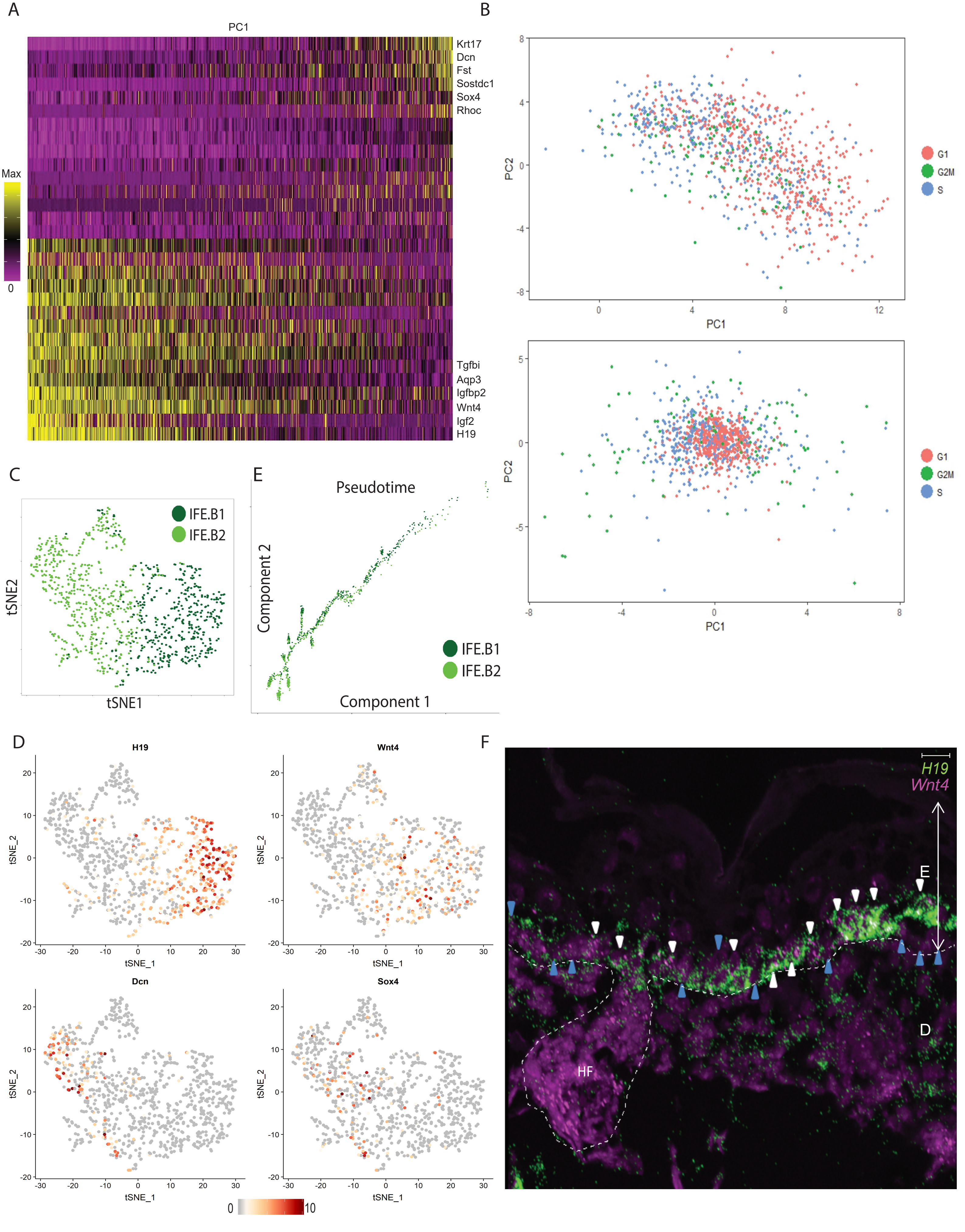

Supplementary Fig. 2. **The P0 interfollicular epidermis (IFE) is composed of two basal cell populations. Related to Figure 2.**

A) A heatmap showing expression of top loading genes in PC1 for PCA analysis of all basal IFE cells. **B**) PCA analysis of all basal IFE cells using cell cycle genes. Inferred cell cycle phases are projected onto the PCA plot prior (top) and after (bottom) the effect of cell cycle genes were regressed out. **C**) tSNE result of all basal IFE cells after cell cycle genes were regressed out. The two basal IFE clusters identified in Fig. 1A are projected onto the plot. **D**) Expression level of the basal subcluster markers *H19* and *Wnt4* (IFE.B1) and *Dcn* and *Sox4* (IFE.B2) projected onto the tSNE plot in panel C. **E**) Pseudotime trajectory of all basal IFE cells with the identity of the two basal subclusters projected onto the trajectory. **F**) RNA-FISH of *H19* (green) and *Wnt4* (purple). White arrowheads point to *Wnt4*-high cells and blue arrows point to *Wnt4*-low cells; high *Wnt4* expression coincides with high *H19* expression. Broken white line traces basal lamina. E, epidermis; D, dermis; HF, hair follicle, representative image for N = 5, Scale bar = 10 µm.

Supplementary Figure 3

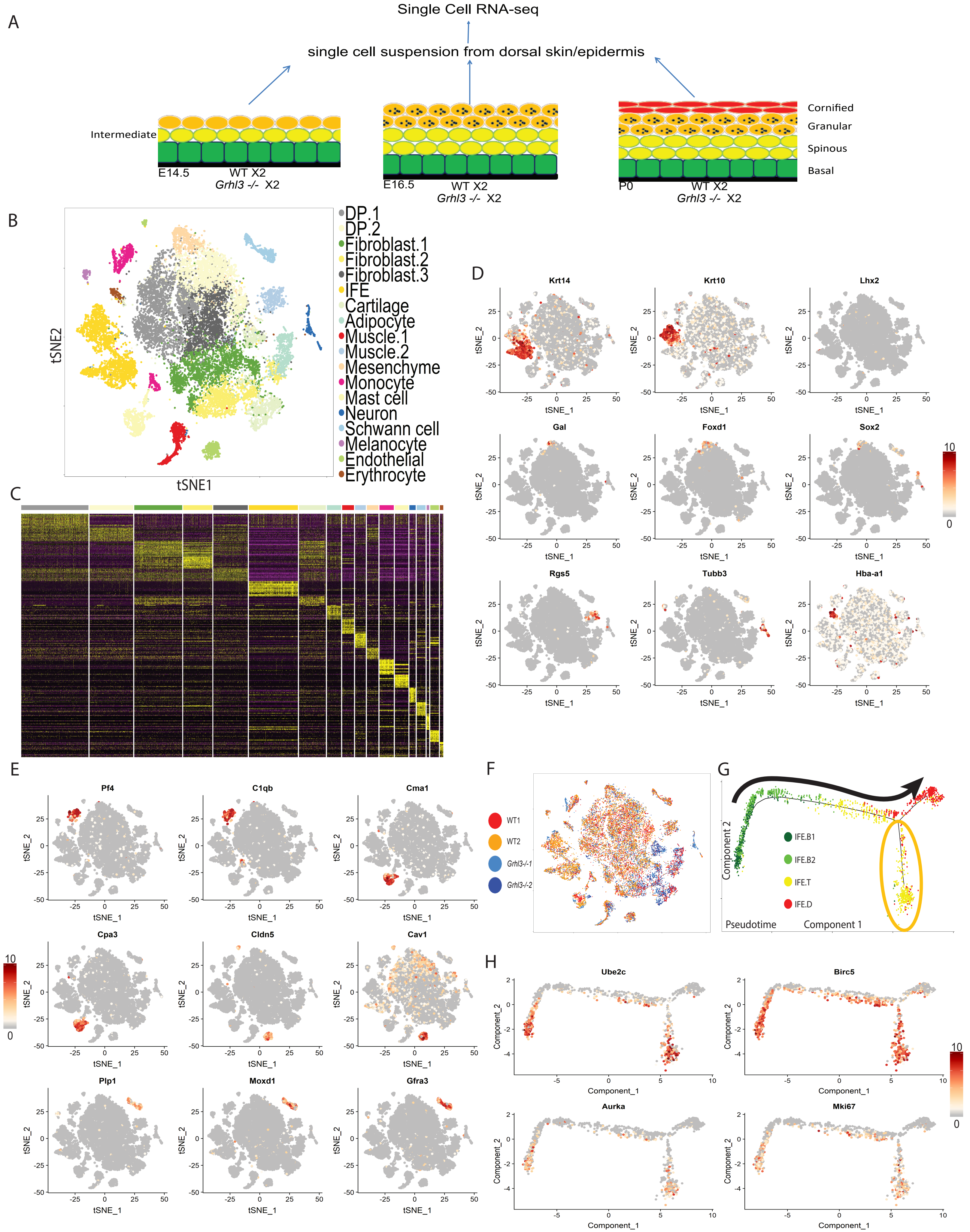

Supplementary Fig. 3. **The IFE differentiation program is established at E14.5 with intermediate layer present. Related to Figure 3.**

A) Experimental scheme for single cell RNA-seq experiments. E14.5, E16.5 skin and P0 epidermis cells in two WT and two *Grhl3*$^{-/-}$ littermates at each time point were collected to generate single cell suspension for scRNA-seq using 10X Genomics Chromium. An additional WT P0 mouse epidermis was also analyzed (Figs. 1-2). **B**) tSNE plot of E14.5 (WT1, WT2, *Grhl3*$^{-/-}$*1, Grhl3*$^{-/-}$*2*) skin cells. **C**) Heatmap showing expression of marker genes for each population in panel A. Gene lists in Supplementary Table 1. **D**) Expression of basal IFE (*Krt14*), suprabasal IFE (*Krt10*), hair follicle (*Lhx2*), mesenchyme (*Gal*, *Foxd1*), astrocyte (*Sox2*), muscle (*Rgs5*) neuron (*Tubb3*) and erythrocyte (*Hba-a1*) markers projected onto tSNE. **E**) Expression level of monocyte (*Pf4*, *C1qb*), mast cell (*Cma1*, *Cpa3*), endothelial cell (*Cldn5*, *Cav1*), and Schwann cell (*Plp1*, *Moxd1*, *Gfra3*) markers projected onto tSNE. **F**) Genotype of each cell projected onto the tSNE plot shown in panel A. **G**) Pseudotime differentiation trajectory of E14.5 IFE cells. The trajectory is consistent with IFE differentiation, going from basal to transition to differentiated cells (arrow); also, the transition population (presumed intermediate layer keratinocytes) forms a branch, apparently due to high cell proliferation; highlighted with the orange circle. **H**) Expression levels of proliferation markers *Ube2c*, *Birc5*, *Aurka*, and *Mki67* projected onto pseudotime trajectory in panel G. IFE.B and IFE.T cells are highly proliferative.

Supplementary Figure 4

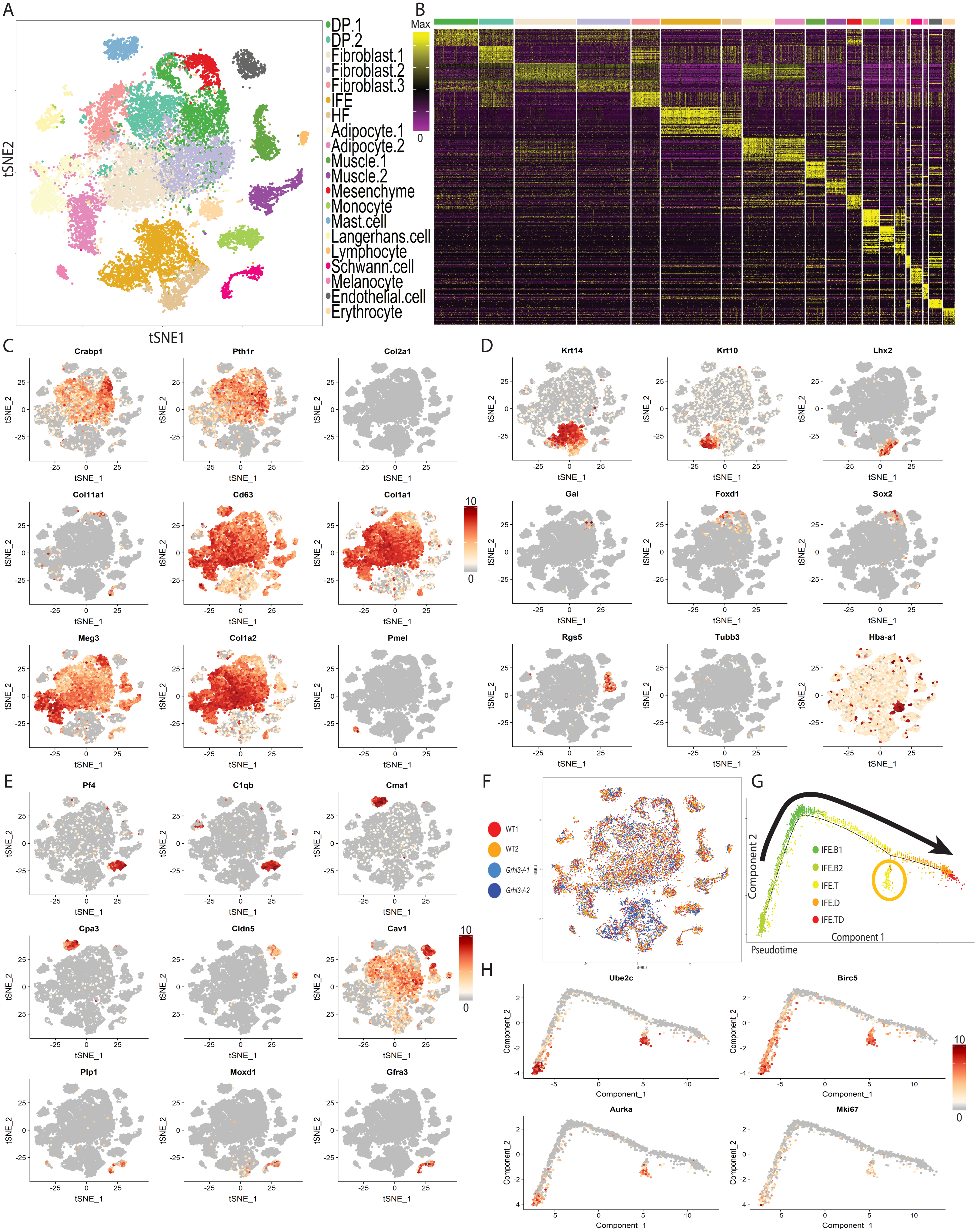

Supplementary Fig. 4. **E16.5 mouse whole skin cellular heterogeneity shows decreased proliferating intermediate layer. Related to Figure 3**

A) tSNE plot of E16.5 (WT1, WT2, *Grhl3*$^{-/-}$*1, Grhl3*$^{-/-}$*2*) skin cell populations. **B**) Heatmap showing expression of marker genes for each population in panel A. Gene lists in Supplementary Table 1. **C**) Expression level of dermal papillae (*Crabp1*, *Pth1r*), cartilage (*Col2a1*, *Col11a1*), dermal fibroblast (*Cd63*, *Col1a1*, *Meg3*, *Col1a2*) and melanocytes (*Pmel*) markers projected onto tSNE. **D**) Expression of basal IFE (*Krt14*), suprabasal IFE (*Krt10*), hair follicle (*Lhx2*), mesenchyme (*Gal*, *Foxd1*), astrocytes (*Sox2*), muscle (*Rgs5*) neuron (*Tubb3*) and erythrocyte (*Hba-a1*) markers projected onto tSNE. **E**) Expression level of monocytes (*Pf4*, *C1qb*), mast cells (*Cma1*, *Cpa3*), endothelial cells (*Cldn5*, *Cav1*), and Schwann cell (*Plp1*, *Moxd1*, *Gfra3*) markers projected onto tSNE. **F**) Genotype of each cell projected onto the tSNE plot shown in panel A. **G**) Pseudotime differentiation trajectory of E16.5 IFE cells. The trajectory is consistent with IFE differentiation, going from basal to transition to differentiated cells (arrow); also, the transition population (presumed intermediate layer keratinocytes) forms a small branch, apparently due to high cell proliferation; highlighted with the orange circle. **H**) Expression level of proliferation markers *Ube2c*, *Birc5*, *Aurka*, and *Mki67* projected onto pseudotime trajectory in panel G. IFE.B and IFE.T cells are highly proliferative.

Supplementary Figure5

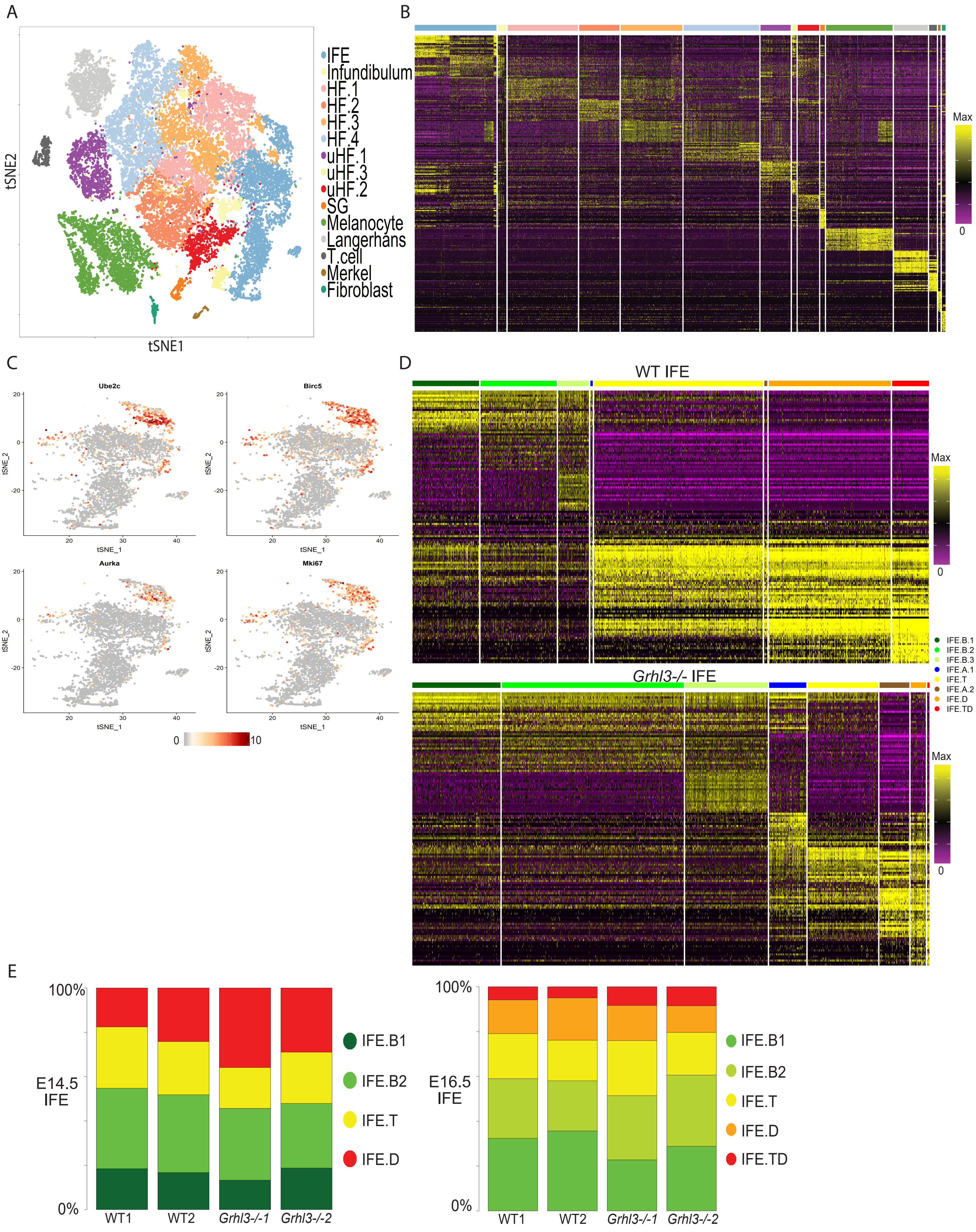

Supplementary Fig. 5. **P0 epidermal heterogeneity shows significant difference in IFE population composition. Related to Figure 4.**

A) tSNE plot of P0 (WT1, WT2, *Grhl3*$^{-/-}$*1, Grhl3*$^{-/-}$*2*) epidermis cell populations. **B**) Heatmap showing expression of marker genes for each population in panel A. Gene lists in Supplementary Table 1. **C**) Expression level of cell proliferation genes *Ube2c, Birc5, Aurka,* and Mki67 projected onto IFE's tSNE plot in Fig. 4A. **D**) Heatmap showing the expression of IFE subpopulation markers in WT (top) and *Grhl3*$^{-/-}$ (bottom) IFE cells. Gene lists in Supplementary Table 1. **E**) Bar charts showing the percentage of each subpopulation in each biological replicate for developmental time points E14.5 and E16.5. The cellular composition is similar in WT and *Grhl3*$^{-/-}$ E14.5 and E16.5 IFE.

Supplementary Figure 6

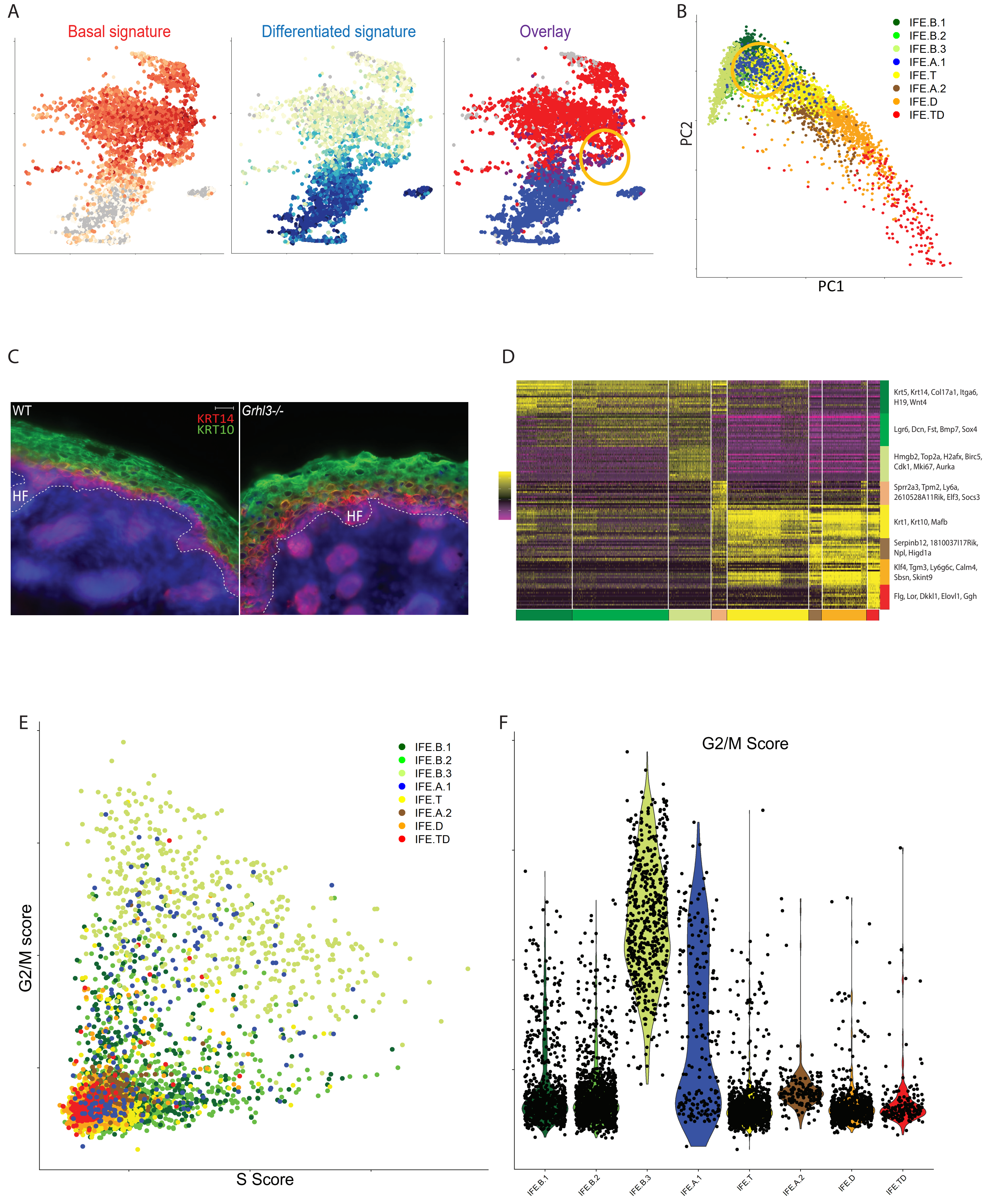

Supplementary Fig. 6. The aberrant IFE.A cell state resembles a transition population and is proliferative. Related to Figure 4.

A) A basal signature score (left) as reflected by the expression levels of *Krt14, Krt5, Col17a1, Itga6,* and *Wnt10a*; a suprabasal signature score (middle) as reflected by the expression levels of *Krt10, Krt1, Krtdap, Klf4,* and *Mafb*; and an overlay of basal and suprabasal signature scores (right) projected onto tSNE plot of P0 IFE (WT and *Grhl3*$^{-/-}$). Orange circle highlights the IFE.A1 population where both basal and suprabasal signature scores are high as indicated in purple. **B**) PCA plot of P0 IFE populations. Orange circle highlight the position of the IFE.A1 population, which is in between the basal and the transition population. **C**) Immunofluorescence of WT and *Grhl3*$^{-/-}$ P0 mouse epidermis against KRT14 and KRT10. A thicker epidermis and the presence of more orange KRT14/KRT10 double positive cells is observed. Representative image for N = 5 each. Scale bar = 10 µm. **D**) Heatmap showing the expression of markers for each IFE cluster in WT and *Grhl3*$^{-/-}$ IFE cells. Sprr2a3, Tpm2, Ly6a etc. are specifically expressed in IFE.A population. Gene lists in Supplementary Table 1. **E**) P0 IFE cells are scored based on the expression of distinct cell cycle genes. G2/M score vs S Score for all P0 IFE cells is shown. A subset of the IFE.A1 population shows high G2/M score, indicative of higher proliferation. **F**) Violin plot of G2/M scores for P0 IFE populations. A subset of IFE.A population shows high G2/M score.

Supplementary Figure 7

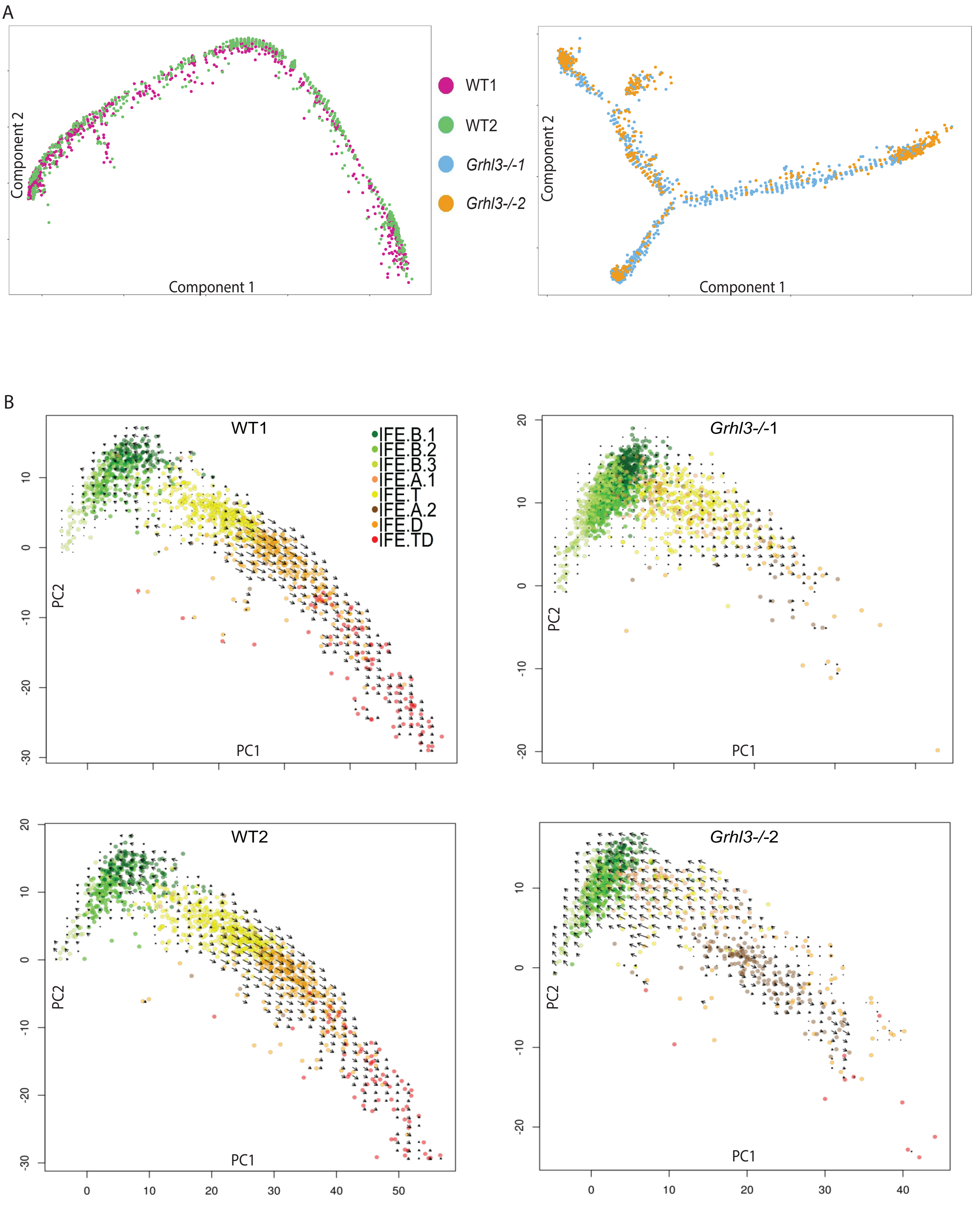

Supplementary Fig. 7. A commitment point separates undifferentiated cells from gradualistically differentiating IFE cells. Related to Figure 5.

A) Genotypes of WT1, WT2, *Grhl3*$^{-/-}$*1*, *Grhl3*$^{-/-}$*2* are projected onto P0 IFE pseudotime trajectories shown in Fig. 5A. **B**) RNA-velocity analysis of WT1, WT2, *Grhl3*$^{-/-}$*1*, *Grhl3*$^{-/-}$*2* using PCA embedding. Note the PCA happens to delineate the differentiation trajectory from basal to transition to differentiated IFE cells. The continuous progression to differentiation shown in WT cells is abolished in the *Grhl3*$^{-/-}$ cells and a propensity of a movement toward basal fate is observed.

Supplementary Figure 8

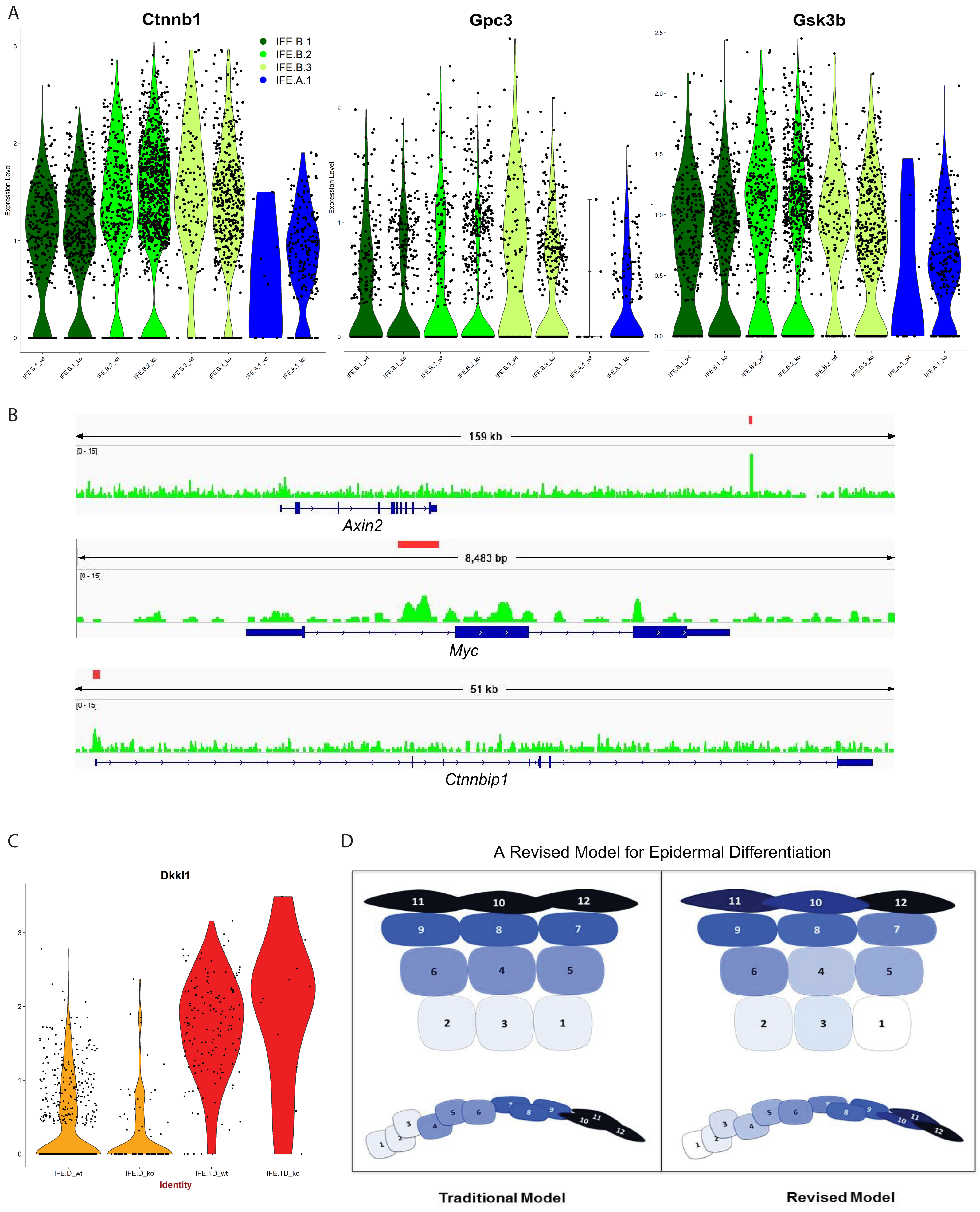

Supplementary Fig. 8. GRHL3 regulates Wnt signaling components. Related to Figures 6 and 7.

A) Violin plots showing the expression of Wnt pathway components *Ctnnb1, Gpc3,* and *Gsk3b* in WT and *Grhl3*$^{-/-}$ basal and IFE.A1 populations. **B**) GRHL3 ChIP-seq signal on the indicated Wnt signaling genes in the E16.5 epidermis. The red bars represent significant peaks. **C**) Violin plots showing the expression of *Dkkl1* in WT and *Grhl3*$^{-/-}$ IFE.D and IFE.TD populations. **D**) Cartoon depicting a model for a gradualistic IFE differentiation process as opposed to the traditional model where differentiation occurs in punctuated stages.
