# Supplementary material for: Murine interfollicular epidermal differentiation is gradualistic with GRHL3 controlling progression from stem to transition cell states
Source: Nat Commun. 2020 Oct 28;11:5434. doi: 10.1038/s41467-020-19234-6 (PMC7595230; doi:10.1038/s41467-020-19234-6)
Supplement: Supplementary file 3 — Description of Additional Supplementary Files [file 41467_2020_19234_MOESM3_ESM.pdf]

Title: Supplementary Software

Description: The code performs integration analysis of IFE cells across all three time points (E14.5, E16.5 and P0), including joint clustering using Seurat and pseudotemporal trajectory analysis using scEpath, and generates results for Figure 3.

Title: Supplementary Data 1

Description: Gene lists related to each figure and cell quality control matrices for single cell RNA-seq experiments.
