# Supplementary material for: Murine interfollicular epidermal differentiation is gradualistic with GRHL3 controlling progression from stem to transition cell states
Source: Nat Commun. 2020 Oct 28;11:5434. doi: 10.1038/s41467-020-19234-6 (PMC7595230; doi:10.1038/s41467-020-19234-6)
Supplement: Supplementary file 6 — Reporting Summary [file 41467_2020_19234_MOESM6_ESM.pdf]

nature research

Corresponding author(s): *Double-blind peer review submissions: write DBPR and your manuscript number here instead of author names.*

Last updated by author(s): *YYYY-MM-DD*

Reporting Summary

Nature Research wishes to improve the reproducibility of the work that we publish. This form provides structure for consistency and transparency in reporting. For further information on Nature Research policies, see our Editorial Policies and the Editorial Policy Checklist.

Statistics

For all statistical analyses, confirm that the following items are present in the figure legend, table legend, main text, or Methods section.

n/a	Confirmed	
[ ]	[x]	The exact sample size (*n*) for each experimental group/condition, given as a discrete number and unit of measurement
[ ]	[x]	A statement on whether measurements were taken from distinct samples or whether the same sample was measured repeatedly
[ ]	[x]	The statistical test(s) used AND whether they are one- or two-sided<br>*Only common tests should be described solely by name; describe more complex techniques in the Methods section.*
[x]	[ ]	A description of all covariates tested
[x]	[ ]	A description of any assumptions or corrections, such as tests of normality and adjustment for multiple comparisons
[ ]	[x]	A full description of the statistical parameters including central tendency (e.g. means) or other basic estimates (e.g. regression coefficient) AND variation (e.g. standard deviation) or associated estimates of uncertainty (e.g. confidence intervals)
[ ]	[x]	For null hypothesis testing, the test statistic (e.g. *F*, *t*, *r*) with confidence intervals, effect sizes, degrees of freedom and *P* value noted<br>*Give P values as exact values whenever suitable.*
[x]	[ ]	For Bayesian analysis, information on the choice of priors and Markov chain Monte Carlo settings
[x]	[ ]	For hierarchical and complex designs, identification of the appropriate level for tests and full reporting of outcomes
[x]	[ ]	Estimates of effect sizes (e.g. Cohen's *d*, Pearson's *r*), indicating how they were calculated

Our web collection on statistics for biologists contains articles on many of the points above.

Software and code

Policy information about availability of computer code

Data collection	Illumina HiSeq 4000
Data analysis	Cellranger v2.01, Seurat v1.3, Seurat v2.3.4, Seurat v3.0.1, SC3 v3.11, Monocle v2.10.1, PHATE v0.3.0, scEpath v1.0.0, RNA-Velocity v0.6

For manuscripts utilizing custom algorithms or software that are central to the research but not yet described in published literature, software must be made available to editors and reviewers. We strongly encourage code deposition in a community repository (e.g. GitHub). See the Nature Research guidelines for submitting code & software for further information.

Data

Policy information about availability of data

All manuscripts must include a data availability statement. This statement should provide the following information, where applicable:

- Accession codes, unique identifiers, or web links for publicly available datasets
- A list of figures that have associated raw data
- A description of any restrictions on data availability

All data is availalbe on GEO, GSE154579

Field-specific reporting

Life sciences study design

All studies must disclose on these points even when the disclosure is negative.

Sample size	No sample size calculation was performed, 2 littermate biological samples for each time point and each condition were chosen for scRNA-seq experiments to ensure reproducibility. This sample size was sufficient because in scRNA-seq many individual cells are sampled for each biological sample.
Data exclusions	No data were excluded from the study.
Replication	2 littermate biological replicates for each time point and each condition for all scRNA-seq experiments; all attempts at replication were successful
Randomization	N/A because samples were grouped based on their genotypes, covariates were controlled because they were littermate biological samples.
Blinding	N/A because samples were grouped based on their genotypes

Reporting for specific materials, systems and methods

We require information from authors about some types of materials, experimental systems and methods used in many studies. Here, indicate whether each material, system or method listed is relevant to your study. If you are not sure if a list item applies to your research, read the appropriate section before selecting a response.

Materials & experimental systems

n/a	Involved in the study	
☐	☒	Antibodies
☒	☐	Eukaryotic cell lines
☒	☐	Palaeontology and archaeology
☐	☒	Animals and other organisms
☒	☐	Human research participants
☒	☐	Clinical data
☒	☐	Dual use research of concern

Methods

n/a	Involved in the study	
☒	☐	ChIP-seq
☒	☐	Flow cytometry
☒	☐	MRI-based neuroimaging

Antibodies

Antibodies used	Krt14 Abcam Ab7800, Krt10 Covance PRB-159P, Alexa Fluor 488 Abcam Ab150077, Alexa Fluor 594 Abcam Ab150116
Validation	https://www.abcam.com/cytokeratin-14-antibody-ll002-ab7800.pdf<br>https://www.biolegend.com/en-us/products/keratin-10-polyclonal-antibody-purified-10952<br>https://www.abcam.com/goat-rabbit-igg-hl-alexa-fluor-488-ab150077.html<br>https://www.abcam.com/goat-mouse-igg-hl-alexa-fluor-594-ab150116.html

Animals and other organisms

Policy information about studies involving animals; ARRIVE guidelines recommended for reporting animal research

Laboratory animals	Mus musculus, C57BL/6, Wild type and Grhl3 -/- littermates at age E14.5, E16.5 and P0, 1 male and 1 female for each genotype and each time point were used. Mice used were described in Yu, Z. et al. The Grainyhead-like epithelial transactivator Get-1/Grhl3 regulates epidermal terminal differentiation and interacts functionally with LMO4. Dev. Biol. 299, 122–136 (2006)
Wild animals	No wild animals were used
Field-collected samples	No field collected samples were used
Ethics oversight	All animal experiments were performed in accordance with Institutional Animal Care and Use Committee at University of California, Irvine (Protocol No. AUP-19-012)

Note that full information on the approval of the study protocol must also be provided in the manuscript.
